# Successful treatment of eosinophilic cellulitis with a short course of Dupilumab^☆^

**DOI:** 10.1016/j.abd.2024.07.010

**Published:** 2025-01-15

**Authors:** Hiram Larangeira de Almeida Junior, Ana Letícia Boff

**Affiliations:** a Postgraduate Program in Health and Behavior, Universidade Católica de Pelotas, Pelotas, RS, Brazil; b Santa Casa de Porto Alegre, Porto Alegre, RS, Brazil

*Dear Editor,*

Eosinophilic diseases comprise a heterogeneous group of diseases characterized by tissue eosinophilia that may be accompanied by peripheral eosinophilia.^1^, ^2^ Eosinophilic cellulitis (Wells’ syndrome) is a well known condition which can be mistaken for bacterial infections,^3^, ^4^ and histopathology is essencial for its definitive diagnosis.^1^ Other primary eosinophilic diseases are granuloma faciale, eosinophilic fasciitis (Shulman syndrome), and eosinophilic folliculitis (Ofuji disease).^2^

Histopathology varies according to disease stage: in the acute phase there are more eosinophils; in the subacute phase, histiocytes and “flame figures” are well evident and in the chronic phase a granulomatous reaction predominates.^1^ Flame figures occur due to the adhesion of eosinophilic degranulation material to collagen, but are not always present^2^, ^5^ and occur in other conditions such as pemphigoid, Churg-Strauss syndrome, herpes gestationis, eczema, prurigo, drug-related eruptions, and follicular mucinosis. Clinical and laboratory correlation is necessary for the diagnosis.^1^

The present report describes a 50-year-old female patient, a biochemist, who presented with an erythematous infiltrated painful plaque on her left leg of eight weeks duration (Fig. 1). Histopathology of an incisional biopsy revealed a diffuse inflammatory infiltrate and edema in the upper dermis (Fig. 2A), extending to the dermal-hypodermal junction (Fig. 2B). On high power many eosinophils were evident (Fig. 3) and the diagnosis of eosinophilic cellulitis was made.

**Figure 1:**
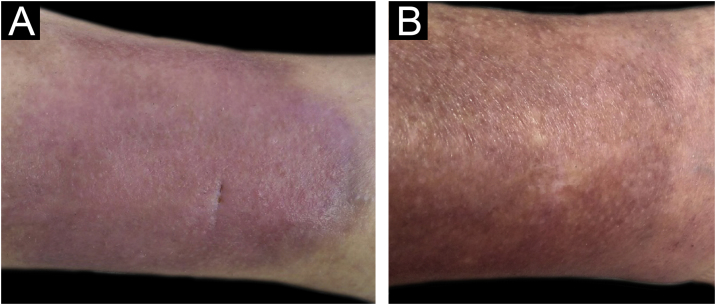
(A) Erythematous infiltrated plaque on the left leg. (B) Appearance after treatment, with residual hyperpigmentation.

**Figure 2:**
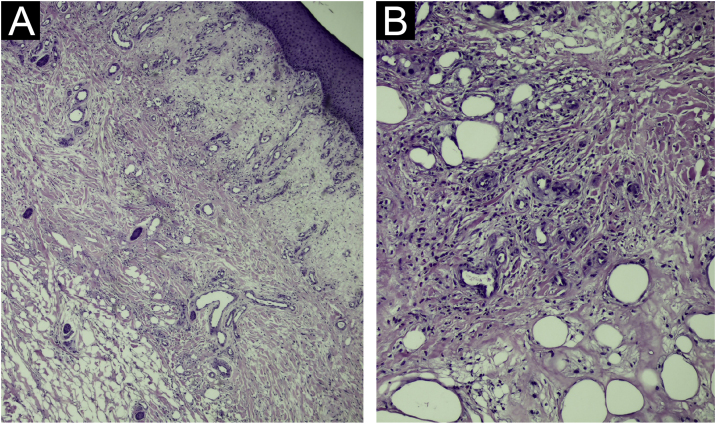
Histopathology: (A) Diffuse inflammatory infiltrate with edema in the upper dermis (Hematoxylin & eosin, ×100). (B) The infiltrate extends to the dermal-hypodermal junction (Hematoxylin & eosin, ×200).

**Figure 3:**
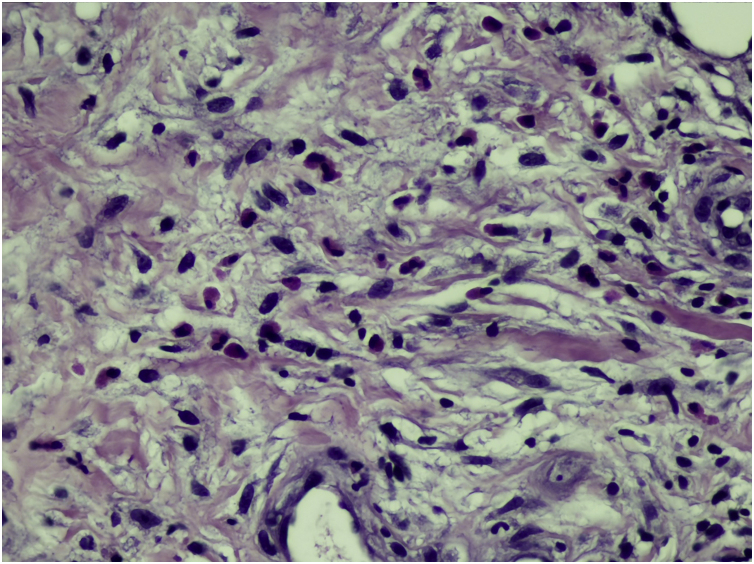
Histopathology: high magnification showing numerous eosinophils (Hematoxylin & eosin, ×400).

The patient was treated with oral prednisone, at an initial dose of 40 mg, together with topical tacrolimus 0.1%. There was a good initial response, with decrease in pain and erythema. Recurrence occurred after the gradual withdrawal of prednisone. Due to refractoriness to previous interventions, subcutaneous dupilumab was started, with a 600 mg loading dose followed by two doses of 300 mg, with a 15-day interval. The treatment, therefore, consisted of only four 300 mg syringes, as this was the available quantity since the patient had financial difficulties to acquire a larger quantity of the medication.

There was a gradual decrease in erythema and pain in the subsequent weeks, and there was no recurrence after ten months of follow-up. Although there was residual hyperpigmentation (Fig. 1) there were no other side effects to the treatment.

The standard treatment for eosinophilic cellulitis is systemic corticosteroids, but recurrence is frequently observed when they are discontinued.^6^ Other medications reported to be effective include methotrexate,^7^ colchicine, dapsone, hydroxychloroquine, and azathioprine.^2^

Dupilumab has already proven effective in several eosinophil-mediated diseases, such as eosinophilic esophagitis,^8^ with four reports of its use in eosinophilic cellulitis, three in the United States^6^, ^9^, ^10^ and one in Germany,^11^ all of which were patients resistant to conventional therapy. Interestingly, one case in the United States was treated with only four doses, due to financial difficulties in obtaining the drug, and with doses of 200 mg (400 mg loading dose followed by two doses of 200 mg, every 15 days), with a response similar to the case described herein.^9^

Other immunobiologicals have also been successfully reported, such as omalizumab (anti-IgE) and mepolizumab (anti-IL-5), as well as adalimumab, an anti-TNF-α.^1^

Dupilumab targets the IL-4 alpha receptor, interfering with IL-4 and IL-13 signaling involved in eosinophil activation.^9^ This is a relevant alternative in the treatment of diseases mediated by this cell. Possibly, short treatments may be sufficient to interrupt its activation in the skin.

## Financial support

None declared.

## Authors’ contributions

Hiram Larangeira de Almeida Jr: Approval of the final version of the manuscript; design and planning of the study; drafting and editing of the manuscript; collection, analysis, and interpretation of data; effective participation in research orientation; intellectual participation in the propaedeutic and/or therapeutic conduct of the studied cases; critical review of the literature; critical review of the manuscript.

Ana Letícia Boff: Approval of the final version of the manuscript; design and planning of the study; drafting and editing of the manuscript; collection, analysis, and interpretation of data; intellectual participation in the propaedeutic and/or therapeutic conduct of the studied cases; critical review of the literature; critical review of the manuscript.

## Conflicts of interest

None declared.
